# Effect of sildenafil on the activity of some antidepressant drugs and electroconvulsive shock treatment in the forced swim test in mice

**DOI:** 10.1007/s00210-016-1334-3

**Published:** 2016-12-24

**Authors:** Katarzyna Socała, Dorota Nieoczym, Elżbieta Wyska, Piotr Wlaź

**Affiliations:** 1 Department of Animal Physiology, Institute of Biology and Biochemistry, Faculty of Biology and Biotechnology, Maria Curie-Skłodowska University, Akademicka 19, PL-20033 Lublin, Poland; 2 Department of Pharmacokinetics and Physical Pharmacy, Collegium Medicum, Jagiellonian University, Kraków, Poland

**Keywords:** Sildenafil, Antidepressant drugs, Electroconvulsive treatment, Forced swim test

## Abstract

Sildenafil, a potent and selective inhibitor of phosphodiesterase type 5, is used clinically to treat erectile dysfunction and pulmonary arterial hypertension. It is often taken by patients suffering from depression and receiving antidepressant drug treatment. However, its influence on the efficacy of antidepressant treatment was not sufficiently studied. Therefore, the aim of the present study was to investigate the influence of sildenafil on the anti-immobility action of several antidepressant drugs (i.e., sertraline, fluvoxamine, citalopram, maprotiline, trazodone, and agomelatine) as well as on antidepressant-like effect of electroconvulsive stimulations in the forced swim test in mice. The obtained results showed that acute sildenafil treatment enhanced the antidepressant-like activity of all of the studied drugs. The observed effects were not due to the increase in locomotor activity. The interactions between sildenafil and sertraline, maprotiline, and trazodone were pharmacodynamic in nature, as sildenafil did not affect concentrations of these drugs neither in serum nor in brain tissue. Increased concentrations of fluvoxamine, citalopram, and agomelatine in brain tissue evoked by sildenafil co-administration suggest that pharmacokinetic interactions between sildenafil and these drugs are very likely. Sildenafil injected acutely did not alter the antidepressant-like efficacy of electroconvulsive stimulations in mice, as assessed in the forced swim test. Interestingly, repeated (14 days) administration of sildenafil decreased the anti-immobility action of the electroconvulsive stimulations. In conclusion, the present study shows that sildenafil may alter the effectiveness of antidepressant treatment. Further studies are warranted to better characterize the influence of sildenafil on the activity of antidepressant drugs and electroconvulsive therapy.

## Introduction

Major depressive disorder, also known as major depression, is the most common mental disorder that is the leading cause of disability worldwide. In the USA, the total economic burden of major depression was estimated to be $210.5 billion per year (Greenberg et al. 2015). Antidepressant drugs, first introduced in the 1950s, remain the mainstay of treatment of depression and are one of the most widely taken medicines (Lopez-Munoz and Alamo 2009). High rates of comorbidity between depression and various somatic and/or mental illnesses result in a need for simultaneous treatment with antidepressants and other medications. This requires special caution, especially concerning drug interactions (Kang et al. 2015; Lopez-Munoz and Alamo 2009; Iosifescu et al. 2004). For severe and life-threatening depression that does not respond to antidepressants drugs, electroconvulsive therapy (ECT) is frequently used (Payne and Prudic 2009).

Various studies have shown that depression often co-occurs with erectile dysfunction. The incidence of these two conditions ranges from 18 to 35%. In severe depression, erectile dysfunction may be as high as 90% (Farre et al. 2004). Sildenafil citrate, a selective and competitive inhibitor of phosphodiesterase type 5 (PDE5), is the first-line oral treatment for erectile dysfunction (Rosen and McKenna 2002). It was shown to be effective in men with co-occurring depression and erectile dysfunction, including those taking antidepressants (Nurnberg et al. 2002; Seidman et al. 2001). Sildenafil has also a well-proven efficacy against erectile dysfunction evoked by antidepressant therapy, mainly with selective serotonin reuptake inhibitors (SSRIs) (Nurnberg and Hensley 2003). Men suffering from depression and erectile dysfunction are not the only group of patients who may be taking antidepressants and sildenafil. Of note, sildenafil was reported to reduce antidepressant-associated sexual dysfunction in women (Nurnberg et al. 2008). Moreover, sildenafil is one of the management strategies for the treatment of pulmonary arterial hypertension (Wang et al. 2014) that is a rare and debilitating disease with concomitant depressive symptoms estimated at ~55% (McCollister 2011).

Although sildenafil is often used in patients treated with antidepressant drugs, there is no clear-cut evidence that it augments or worsens antidepressant action in humans. In clinical trials evaluating the efficacy of sildenafil against antidepressant-associated erectile dysfunction, mean depression scores were consistent with remission evoked by antidepressant therapy in both placebo- and sildenafil-treated groups (Nurnberg and Hensley 2003; Nurnberg et al. 2003). Data obtained from animal studies are, however, quite controversial. Since sildenafil works via the nitric oxide/cyclic guanosine 3′,5′-monophosphate (NO/cGMP) pathway (Rosen and McKenna 2002), it is often used as a pharmacological tool to study the role of this signaling pathway in the mechanism of action of various compounds, including antidepressants. For example, sildenafil was shown to abolish the antidepressant-like effect of memantine (Almeida et al. 2006), lithium (Ghasemi et al. 2008), escitalopram (Zomkowski et al. 2010), duloxetine (Zomkowski et al. 2012), and paroxetine (Socała et al. 2012d) in the forced swim test in mice, whereas it failed to reverse the antidepressant effect of fluoxetine in rats (Brink et al. 2008). Interestingly, sildenafil at a relatively small dose (5 mg/kg) reversed the antidepressant action of bupropion (Dhir and Kulkarni 2007b) and venlafaxine (Dhir and Kulkarni 2007a), while in another study, when injected at higher doses (10–20 mg/kg), it was reported to enhance the anti-immobility effect of bupropion and venlafaxine in mice (Socała et al. 2012a). Furthermore, sildenafil caused a potent enhancement of the activity of amitriptyline (a tricyclic antidepressant that exhibits cholinolytic properties), but without affecting the activity of desipramine—a tricyclic antidepressant devoid of antimuscarinic action (Socała et al. 2012c). The anti-immobility action of two atypical antidepressants, mianserin and tianeptine, was also enhanced by sildenafil co-administration (Socała et al. 2012b). It is worth noting that the sildenafil-induced potentiation of the antidepressant-like effects of bupropion, venlafaxine, and tianeptine might have been, at least in part, due to pharmacokinetic interactions between these drugs and sildenafil (Socała et al. 2012a, b).

Thus, sildenafil appears to exert various effects on the activity of antidepressant drugs depending on the mechanism of action of the respective drug, the dose of sildenafil used, and/or other yet unidentified factors. It seems that the interactions between sildenafil and antidepressants should be estimated for each drug individually. Therefore, the aim of the present study was to evaluate the influence of sildenafil on the activity of other antidepressant drugs (i.e., sertraline, fluvoxamine, citalopram, maprotiline, trazodone, and agomelatine) in the forced swim test in mice. In addition, we aimed to investigate the impact of sildenafil on the antidepressant effect of the repeated electroshock stimulation (ECS) which mimics the ECT therapy in humans. Moreover, plasma and brain concentrations of antidepressant drugs were measured to determine any pharmacokinetic contribution to the observed behavioral effects.

## Materials and methods

### Animals

Experimentally naïve male albino Swiss mice weighing 25–30 g were used in all experiments. The animals were purchased from a licensed breeder (Laboratory Animals Breeding, Ilkowice, Poland) and housed in groups of eight in Makrolon cages (37 cm × 21 cm × 14 cm) under strictly controlled laboratory conditions (temperature maintained at 21–24 °C, relative humidity 45–65%) with an artificial 12/12-h light/dark cycle (lights on at 6:00 a.m.). A nutritionally balanced rodent chow diet (Murigran; Agropol S.J., Motycz, Poland) and tap water were continuously available. Animals were used in the study after at least 1 week of acclimatization. All experiments were performed between 8:00 a.m. and 2:00 p.m., after a minimum 30-min acclimatization period to the conditions kept in the experimental room. The animals were randomly assigned to the experimental groups. Each animal was used only once.

The study was performed under experimental protocols approved by the Ethical Committee of the Medical University in Lublin. Housing and experimental procedures were conducted in accordance with the European Union Directive of 22 September 2010 (2010/63/EU) and Polish legislation acts concerning animal experimentation. All efforts were made to minimize animal suffering as well as the number of animals used in the study.

### Drugs and treatments

Sildenafil citrate (kindly provided by Polpharma S.A., Starogard Gdański, Poland) was dissolved in a 1% aqueous solution of Tween 80 (POCH, Gliwice, Poland), citalopram hydrobromide (Cipramil; Lundbeck GmbH, Hamburg, Germany) and trazodone hydrochloride (Sigma-Aldrich, St. Louis, MO, USA) were dissolved in normal saline, whereas sertraline hydrochloride (Miravil; Orion Pharma, Espoo, Finland), fluvoxamine maleate (Fevarin; Abbott Healthcare SAS, Châtillon-sur-Chalaronne, France), maprotiline hydrochloride (Ludiomil; Amdipharm Limited, Dublin, Ireland), and agomelatine (Valdoxan; Servier, Suresnes, France) were suspended in a 0.5% aqueous solution of methyl cellulose (Sigma, Steinheim, Germany). The pretreatment time for sildenafil, citalopram, trazodone, and maprotiline was 30 min, while sertraline, fluvoxamine, and agomelatine were injected 60 min before the tests. In studies dealing with the effect of sildenafil on the repeated ECS procedure, sildenafil was administered acutely 30 min before the behavioral tests or repeatedly every 24 h for 14 consecutive days. In the subchronic studies, all experiments were performed 24 h after the last injection in order to avoid acute effects of the drug on animal behavior (Dadomo et al. 2013). All drug solutions/suspensions were prepared freshly and administered intraperitoneally (i.p.) at a volume of 0.1 ml per 10 g of body weight. Control animals received a combination of respective vehicles. In studies assessing the effect of repeated (14 days) sildenafil treatment on the efficacy of the ECS procedure, animals from the sham (control) group and from the ECS-treated group were injected with vehicle repeatedly every 24 h for 14 consecutive days.

The doses and pretreatment schedules were based on those reported in the literature and on previous experiments in our laboratory. The initial dose for sildenafil treatment was 20 mg/kg. The dose was then increased or decreased depending on the observed effect. To avoid repetition, the effect of sildenafil administered alone was not investigated in the present study.

### Electroconvulsive stimulation

Electroconvulsive stimuli (0.75-ms duration pulses at 100 Hz for 0.5 s, current intensity 10–16 mA) were delivered via saline-soaked corneal electrodes using a Grass model CCU1 constant current unit coupled to a Grass S48 stimulator (Grass Technologies, Warwick, RI, USA). Before stimulation, the corneal electrodes were wetted with saline to provide good electrical contact. The criterion for successful ECS was the presence of generalized tonic–clonic convulsions lasting for 5–10 s. To prevent death due to respiratory failure, mice were placed to a box filled with pure oxygen for 3 min before the ECS and immediately after the ECS. Despite oxygen supply, several mice died after seizure. Mice from the sham (control) group were subjected to the same procedures but without delivering the electric current. The animals were subjected to the ECS every other day for 2 weeks, with a total of seven treatments. Behavioral tests were performed 24 h after the last ECS.

### The forced swim test

The test procedure was performed following the method described by Porsolt et al. (1977). Mice were placed individually into glass cylinders (height 25 cm, diameter 10 cm) containing 11 cm of water maintained at a temperature of 23–25 °C. Animals were allowed to swim for 6 min. After the initial 2 min of vigorous activity, the total duration of immobility was recorded during the last 4 min of the test. Mice were considered immobile when they stopped struggling, remained floating passively, made no attempts to escape, and showed only slow limb movements necessary to keep its head above the water. Water in the beakers was changed regularly between subjects. The immobility time was recorded by a trained observer who was aware of the nature of the treatment, with the help of cumulative stopwatches. Data obtained in groups of 10–12 mice were expressed as means (in s) ± standard error of the mean (SEM).

### The locomotor activity test

To monitor the spontaneous locomotor activity of mice, an IR Actimeter system (Panlab/Harvard Apparatus, Barcelona, Spain) was used. This consisted of a square arena (25 × 25 cm) surrounded by a frame containing a total of 16 × 16 infrared beams located on the sides. The frame was coupled to a computerized control unit. Mice were placed individually in the actimeter, in which they were allowed to explore freely for 6 min. The arena was cleaned thoroughly with a 0.1% acetic acid solution before each mouse was placed in it. Locomotor activity was defined as a horizontal activity with displacement and was expressed in terms of a total number of interruptions of the photo beams within the last 4 min of the test, which corresponds to the time interval analyzed in the forced swim test. Interruptions of the photo beams were recorded automatically and analyzed with a computerized system (SedaCom32; Panlab/Harvard Apparatus, Barcelona, Spain). Data obtained in groups of 9–12 mice were expressed as means of activity counts/4 min ± SEM.

### Determination of antidepressant concentrations

Thirty minutes following administration of citalopram, maprotiline, and trazodone and 60 min following administration of sertraline, fluvoxamine, and agomelatine with or without sildenafil, mice were decapitated to collect biological material for pharmacokinetic studies. Blood was collected into Eppendorf tubes and allowed to clot at room temperature. Subsequently, it was centrifuged at 5600 rpm for 10 min and serum was collected into polyethylene tubes. Immediately after the decapitation, brains were dissected from the skull and washed with 0.9% NaCl. The samples were kept at −25 °C until analysis.

Serum and brain concentrations of all antidepressants studied were measured by a high-performance liquid chromatography (HPLC) method. The brains were homogenized in distilled water (1:4, *w*/*v*) with a tissue homogenizer TH220 (Omni International, Inc., Warrenton, VA, USA). The extraction of most drugs studied from serum and brain homogenates was performed using the ethyl acetate/hexane (30:70, *v*/*v*) mixture. Paroxetine (200 ng/ml) was used as an internal standard (IS) for trazodone, citalopram, and sertraline; trazodone (500 ng/ml) for maprotiline; desipramine (250 ng/ml) for fluvoxamine; and carbamazepine (100 ng/ml) for agomelatine. In order to isolate citalopram, fluvoxamine, maprotiline, sertraline, and trazodone, to serum (50–100 μl) or brain homogenate (100–250 μl) containing these drugs, an appropriate IS was added and the samples were alkalized with 50 μl of 4 M NaOH. Then, the samples were extracted with 1 ml of the extraction reagent by shaking for 20 min (IKA Vibrax VXR, Germany). After centrifugation for 4 min (Abbott Diagnostics Division TDX/TM centrifuge), the organic layers were transferred to new Eppendorf tubes containing a 100 μl solution of 0.1 M H_2_SO_4_ and methanol (90:10, *v*/*v*), shaken for 20 min, and then centrifuged for 4 min. The organic layers were discarded, and the aliquots (10 μl) of acidic solutions were injected into the HPLC system.

Agomelatine was extracted with 1 or 5 ml of dichloromethane as the extraction reagent from 200 μl of serum or 1 ml of brain homogenate, respectively, after the addition of IS. In order to precipitate proteins, 1 ml of the concentrated NaCl solution (10 g/50 ml) was added to brain homogenate before the extraction and the samples were vortexed for 15 s. Then, both serum and homogenate samples were shaken for 20 min (IKA Vibrax VXR, Germany) and centrifuged for 15 min at 3000 rpm (Universal 32, Hettich, Germany). Subsequently, the organic layers were transferred into conical glass tubes and evaporated to dryness at 37 °C under a gentle stream of nitrogen in a water bath. The residues were dissolved with 100 μl of methanol, and 50 μl of these solutions was injected into the HPLC system.

The HPLC system (Merck Hitachi, Darmstadt, Germany) consisted of an L-7100 isocratic pump, an L-7200 autosampler, and a UV variable-wavelength K-2600 detector (Knauer, Berlin, Germany). D-7000 HSM software was used for data acquisition and processing. Analyses of most drugs were performed on a 250 × 4.6 mm Supelcosil LC-CN column with a particle size of 5 μm (Sigma-Aldrich, Steinheim, Germany) protected with a guard column (20 × 4 mm) with the same packing material. The only exception was agomelatine which was separated using a 250 × 4 mm LiChrospher^®^ 100 RP-18 column with a particle size of 5 μm (Merck, Darmstadt, Germany) protected with a LiChrospher RP-18 guard column (4 × 4 mm). The mobile phase consisting of acetonitrile and 50 mM potassium dihydrogen phosphate was mixed at a ratio of 21:79 (*v*/*v*) for maprotiline, sertraline, and trazodone; 25:75 (*v*/*v*) for citalopram and fluvoxamine; and 35:65 (*v*/*v*) for agomelatine and was run at 1 ml/min. Chromatographic analyses were carried out at 21 °C and the analytical wavelength was 240 nm for citalopram, 230 nm for agomelatine, and 214 nm for the remaining antidepressants studied.

The calibration curves were constructed by plotting the ratio of the peak heights of the studied drug to IS versus drug concentration, and they were linear in the tested concentration ranges. The assays were reproducible with low intra- and inter-day variation (coefficient of variation less than 10%). The extraction efficiencies of the analyzed compounds and internal standards ranged from 70 to 92%. No interfering peaks were observed in the blank serum or brain homogenate in the regions of interest. Similarly, sildenafil did not interfere with the developed methods. Antidepressant concentrations were expressed in nanograms per milliliter of serum or micrograms per gram of wet brain tissue.

### Statistical analysis

Data obtained in behavioral tests were evaluated by using one-way analysis of variance (one-way ANOVA) followed by Tukey’s post hoc test for multiple comparisons. Concentrations of the tested antidepressant drugs were compared by unpaired Student’s *t* test. Statistical significance was noted when *p* values were equal to or less than 0.05. All calculations were carried out with GraphPad Prism, version 5.03, for Windows (GraphPad Software, San Diego, CA, USA).

## Results

### Effect of sildenafil on the activity of antidepressant drugs in the forced swim test

**Fig. 1:**
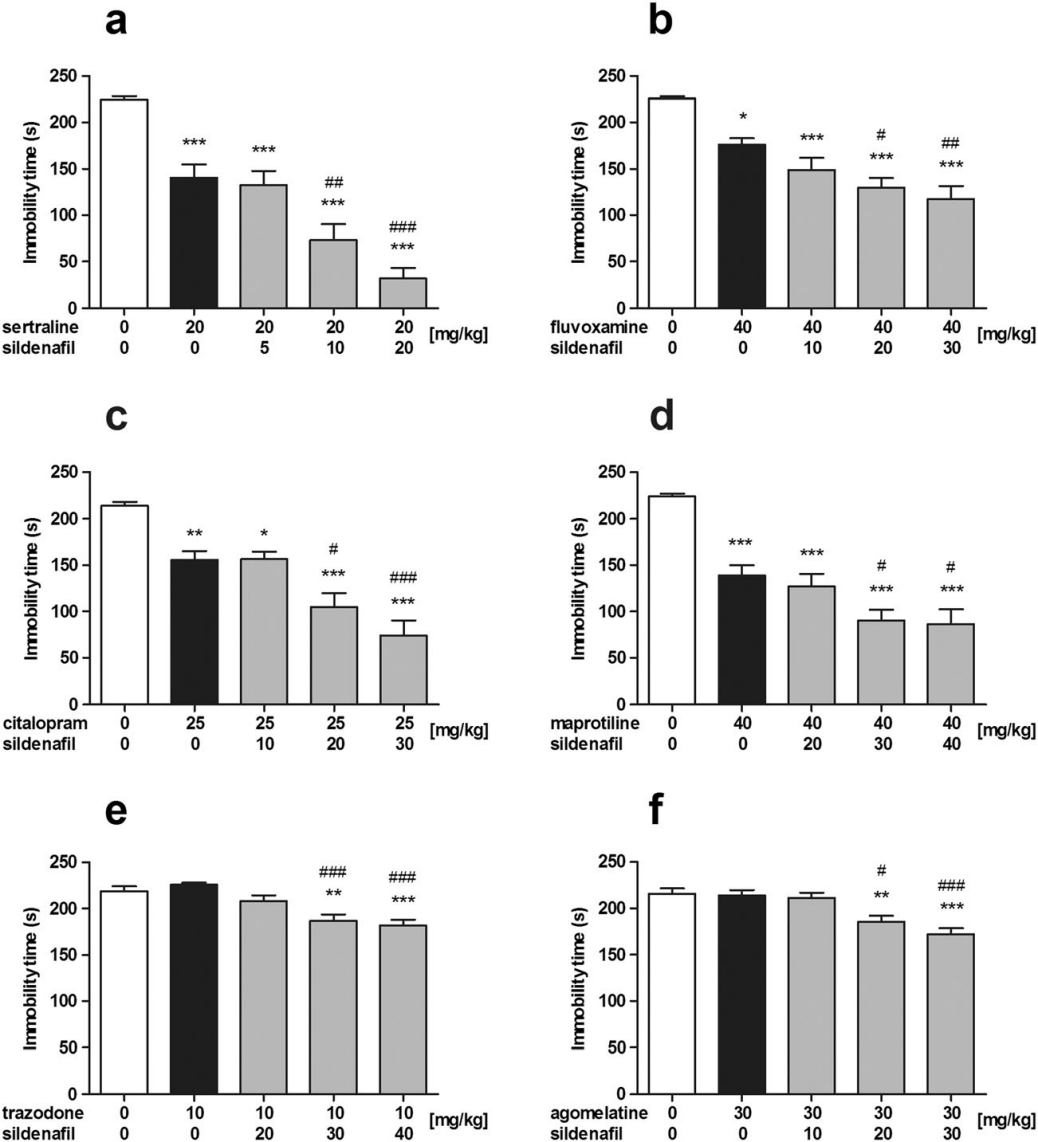
**a**–**f** Effects of antidepressant drugs administered alone and in combination with sildenafil on the immobility time in the forced swim test in mice. Sildenafil, citalopram, maprotiline, and trazodone were injected 30 min before the test, while sertraline, fluvoxamine, and agomelatine 60 min before the test. Control animals received vehicles only. Each experimental group consisted of 10–12 animals. Data are presented as means + SEM. *p < 0.05, **p < 0.01, ***p < 0.001, as compared to the control group; ^#^ *p* < 0.05, ^##^ *p* < 0.01, ^###^ *p* < 0.001, as compared to the antidepressant-treated group (one-way ANOVA followed by Tukey’s post hoc test)

The effect of sildenafil on the antidepressant-like activity of sertraline in the forced swim test in mice is shown in Fig. 1a (one-way ANOVA: *F*(4, 55) = 30.86, *p* < 0.0001). Sertraline administered alone at a dose of 20 mg/kg significantly shortened the total immobility duration as compared to the control group (*p* < 0.001). Co-administration of sertraline and sildenafil at a dose of 10 mg/kg caused ~50% decrease in the immobility time as compared to the sertraline-treated group (*p* < 0.01), while joint administration of sertraline and sildenafil at a dose of 20 mg/kg caused over 75% decrease in immobility time as compared to sertraline given alone (*p* < 0.001). Sildenafil at the lowest dose tested, i.e., 5 mg/kg, did not affect the antidepressant-like effect of sertraline.

The effect of sildenafil on the antidepressant-like activity of fluvoxamine in the forced swim test in mice is shown in Fig. 1b (one-way ANOVA: *F*(4, 52) = 16.05, *p* < 0.0001). Fluvoxamine administered alone at a dose of 40 mg/kg significantly reduced the immobility time as compared to the control group (*p* < 0.05). Sildenafil injected at doses of 20 and 30 mg/kg augmented the anti-immobility action of fluvoxamine by ~30% (*p* < 0.05 and *p* < 0.01 vs. the fluvoxamine-treated group, respectively). Co-administration of fluvoxamine and sildenafil at a dose of 10 mg/kg did not cause any additional effects on the total immobility duration as compared to the fluvoxamine-treated group.

The effect of sildenafil on the antidepressant-like activity of citalopram in the forced swim test in mice is shown in Fig. 1c (one-way ANOVA: *F*(4, 51) = 21.15, *p* < 0.0001). Citalopram administered alone at a dose of 25 mg/kg significantly reduced the total immobility duration as compared to the control group (*p* < 0.01). Sildenafil at the lowest dose tested, i.e., 10 mg/kg, did not affect the anti-immobility effect of citalopram. At a dose of 20 mg/kg, sildenafil significantly enhanced the antidepressant-like activity of citalopram (*p* < 0.05 as compared to the citalopram-treated group). The highest dose of sildenafil (30 mg/kg) caused ~50% increase in the anti-immobility action of citalopram (*p* < 0.001 vs. the citalopram-treated group).

The effect of sildenafil on the antidepressant-like activity of maprotiline in the forced swim test in mice is shown in Fig. 1d (one-way ANOVA: *F*(4, 55) = 21.60, *p* < 0.0001). Maprotiline administered alone at a dose of 40 mg/kg significantly reduced the total immobility duration (*p* < 0.001 vs. the control group). Joint administration of maprotiline and sildenafil at the lowest dose tested, i.e., 20 mg/kg, did not cause any additional effect on the immobility time as compared to the maprotiline-treated group. However, when injected at higher doses (30 and 40 mg/kg), sildenafil augmented the antidepressant-like effect of maprotiline by ~35% (*p* < 0.05 vs. the maprotiline-treated group for both experimental groups).

The effect of sildenafil on the antidepressant-like activity of trazodone in the forced swim test in mice is shown in Fig. 1e (one-way ANOVA: *F*(4, 54) = 11.36, *p* < 0.0001). Trazodone administered alone at a dose of 10 mg/kg as well as trazodone in combination with sildenafil at a dose of 20 mg/kg did not alter the total immobility duration as compared to the control group. However, joint administration of trazodone with sildenafil at doses of 30 and 40 mg/kg produced ~20% reduction in the immobility time as compared to the control group (*p* < 0.01 and *p* < 0.001, respectively) and as compared to the trazodone-treated group (*p* < 0.001 for both experimental groups).

The effect of sildenafil on the antidepressant-like activity of agomelatine in the forced swim test in mice is shown in Fig. 1f (one-way ANOVA: *F*(4, 53) = 10.41, *p* < 0.0001). Neither agomelatine administered alone at a dose of 30 mg/kg nor joint administration of agomelatine and sildenafil at a dose of 10 mg/kg affected the total immobility duration as compared to the control group. Co-administration of agomelatine with sildenafil at a dose of 20 mg/kg reduced the immobility time by ~15% as compared to both agomelatine-treated and control groups (*p* < 0.05 and *p* < 0.01, respectively). Sildenafil injected at a dose of 30 mg/kg caused further (~20%) reduction in the immobility duration (*p* < 0.001 as compared to the agomelatine- and vehicle-treated groups).

### Effect of sildenafil on antidepressant action of repeated ECS in the forced swim test

**Fig. 2:**
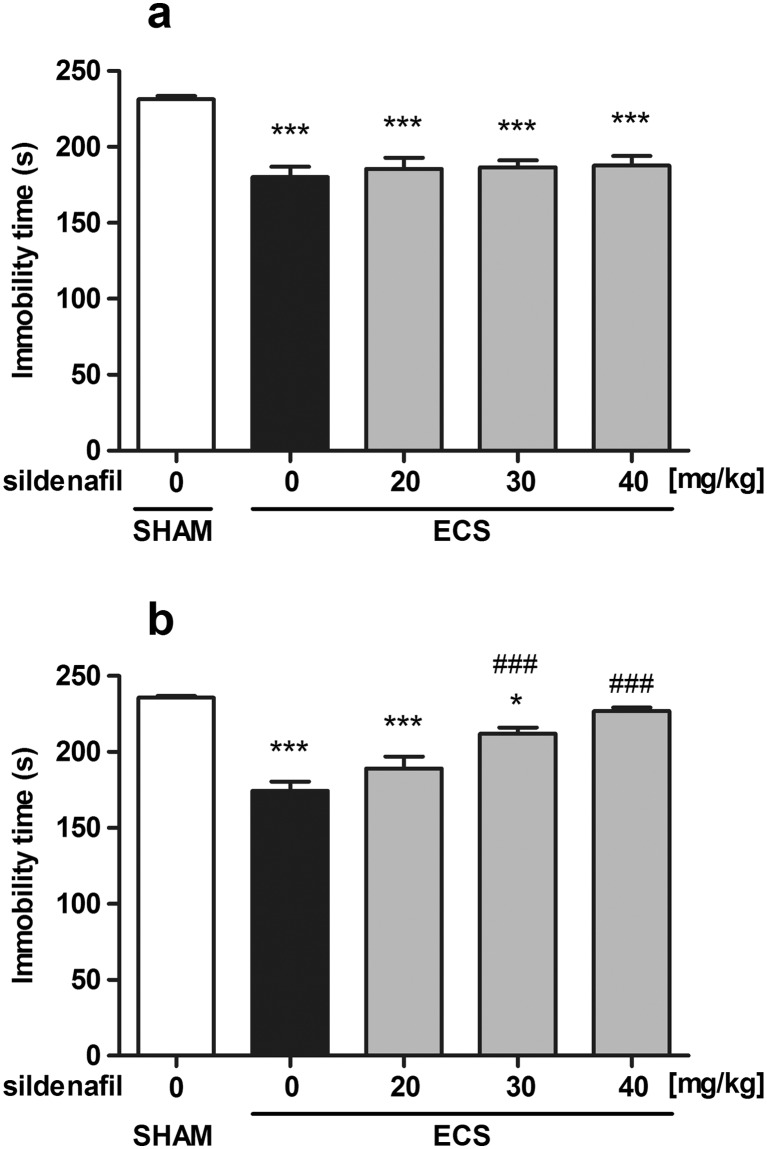
The effect of acute (**a**) and subchronic (**b**) sildenafil treatment on the antidepressant efficacy of the repeated ECS in the forced swim test in mice. In acute studies, sildenafil was administered i.p. 30 min before the test. In subchronic studies, sildenafil was administered once daily for 14 consecutive days. Control and sham animals received vehicle only. Data are presented as means + SEM. Each experimental group consisted of 10–12 animals. *p < 0.05, ***p < 0.001, as compared to the control group; ^###^ *p* < 0.001, as compared to the ECS-treated group (one-way ANOVA followed by Tukey’s post hoc test)

The influence of acute and subchronic treatments with sildenafil on the antidepressant-like effect of the repeated ECS in the forced swim test in mice is shown in Fig. 2 (one-way ANOVA: *F*(4, 51) = 13.80, *p* < 0.0001, in Fig. 2a and *F*(4, 54) = 25.75, *p* < 0.0001, in Fig. 2b). The repeated ECS resulted in a significant anti-immobility effect as compared to the sham group (*p* < 0.001 in both acute and subchronic studies vs. the sham group). Sildenafil administered acutely at doses of 20, 30, and 40 mg/kg did not affect the immobility time as compared to the ECS-treated animals. Subchronic administration of sildenafil at a dose of 20 mg/kg did not affect the anti-immobility effect of the ECS procedure. However, a higher dose of sildenafil (30 mg/kg) significantly weakened the antidepressant-like effect of the ECS (*p* < 0.001 vs. the ECS-treated group; *p* < 0.05 vs. the sham group), while at the highest dose tested (40 mg/kg), sildenafil entirely abolished the anti-immobility action of the ECS procedure (*p* < 0.001 vs. the ECS-treated group).

### Effect of treatments on spontaneous locomotor activity in mice

Data obtained in the spontaneous locomotor activity test in mice is presented in Table 1 (one-way ANOVA: *F*(6, 63) = 8.684, *p* < 0.0001, for a; *F*(6, 60) = 28.22, *p* < 0.0001, for b; *F*(2, 32) = 0.762, *p* = 0.475, for c; and *F*(2, 28) = 6.784, *p* = 0.004 for d). Sertraline, citalopram, and fluvoxamine administered alone as well as in combination with sildenafil did not affect the locomotor activity in mice. Agomelatine administered alone had no significant effect on the activity counts, while agomelatine co-administered with sildenafil reduced the locomotor activity in mice as compared to the control group (*p* < 0.001). Maprotiline and trazodone administered either alone or in combination with sildenafil produced a marked decrease of the locomotor activity (*p* < 0.001 vs. the control group).

**Table 1:** Effect of treatments on the spontaneous locomotor activity in mice

Treatment (mg/kg)	Activity counts/4 min
a.	
Control	1168 ± 170.2
Sertraline (20)	1420 ± 136.8
Sertraline (20) + sildenafil (20)	1173 ± 172.7
Fluvoxamine (40)	1293 ± 140.1
Fluvoxamine (40) + sildenafil (30)	1494 ± 177.7
Agomelatine (30)	826.4 ± 75.1
Agomelatine (30) + sildenafil (30)	279.9 ± 83.2***
b.	
Control	1072 ± 78.7
Citalopram (25)	1326 ± 129.0
Citalopram (25) + sildenafil (30)	1053 ± 187.1
Maprotiline (40)	34.11 ± 12.2***
Maprotiline (40) + sildenafil (40)	48.78 ± 20.3***
Trazodone (10)	430.4 ± 97.0***
Trazodone (10) + sildenafil (40)	121.1 ± 39.6***
c.	
Control	1156 ± 122.3
ECS	1220 ± 114.5
ECS + sildenafil (40), acute	992.9 ± 158.0
d.	
Control	1146 ± 113.3
ECS	749.2 ± 103.2*
ECS + sildenafil (40), subchronic	627.1 ± 87.7**

Citalopram, maprotiline, and trazodone were injected 30 min before the test, while sertraline, fluvoxamine, and agomelatine 60 min before the test. In all acute studies, sildenafil was administered i.p. 30 min before the test. In subchronic studies, sildenafil was administered once daily for 14 consecutive days. All the drugs were injected i.p. Control animals received vehicles only. Data are presented as means ± SEM

*p < 0.05; **p < 0.01; ***p < 0.001, vs. the respective control group (one-way ANOVA followed by Tukey’s post hoc test)

In studies assessing acute effects of sildenafil on the activity of the ECS procedure, no significant alterations in the locomotor activity in mice subjected to the ECS procedure were noticed. Likewise, sildenafil administered acutely to the ECS-treated mice did not produce any significant changes in spontaneous locomotor activity.

Unexpected results were obtained in studies dealing with the influence of subchronic (14 days) sildenafil treatment on the effect of the ECS procedure. Mice subjected to the ECS procedure and vehicle treatment (14 days) as well as mice subjected to the ECS procedure and sildenafil treatment (14 days) exhibited a decreased locomotor activity in comparison to the control group, i.e., injected with vehicle only for 14 days (*p* < 0.05 and *p* < 0.01, respectively).

### Pharmacokinetic studies

The effect of sildenafil on serum and brain concentrations of the tested antidepressant drugs in mice is shown in Table 2. Sildenafil significantly increased brain concentrations of fluvoxamine and citalopram (*p* < 0.001 and *p* < 0.01, respectively) without affecting their concentrations in the serum. Co-administration of sildenafil with agomelatine caused a significant increase in agomelatine concentration in both serum and brain tissue (*p* < 0.05 and *p* < 0.01, respectively). Sildenafil did not produce any changes in the concentrations of sertraline, maprotiline, and trazodone neither in serum nor in brain tissue.

**Table 2:** Effect of sildenafil on the concentrations of antidepressant drugs

Treatment	Drug concentration	
	Serum (ng/ml)	Brain (μg/g)
Sertraline (20)	620.0 ± 77.84	5.90 ± 0.61
Sertraline (20) + sildenafil (20)	477.6 ± 74.42	5.77 ± 0.74
Fluvoxamine (40)	1286 ± 82.38	27.71 ± 2.40
Fluvoxamine (40) + sildenafil (30)	1070 ± 65.57	45.54 ± 2.25***
Citalopram (25)	4471 ± 477.1	32.46 ± 2.31
Citalopram (25) + sildenafil (30)	4291 ± 201.2	46.33 ± 3.93**
Maprotiline (40)	1836 ± 193.6	40.32 ± 1.81
Maprotiline (40) + sildenafil (40)	1894 ± 203.7	39.78 ± 4.31
Trazodone (10)	1186 ± 87.07	1.054 ± 0.15
Trazodone (10) + sildenafil (40)	1074 ± 56.04	1.247 ± 0.16
Agomelatine (30)	44.33 ± 5.68	0.042 ± 0.01
Agomelatine (30) + sildenafil (30)	72.34 ± 12.20*	0.157 ± 0.04**

Sildenafil, citalopram, maprotiline, and trazodone were injected 30 min, while sertraline, fluvoxamine, and agomelatine 60 min before decapitation. Each experimental group consisted of 9–12 animals. Data are presented as means ± SEM

*p < 0.05; **p < 0.01; ***p < 0.001, vs. the respective control group (Student’s *t* test)

## Discussion

Sildenafil was developed as an orally active vasodilator that prolongs the effect of cGMP by selectively inhibiting PDE5 in blood vessels. Its action is not limited only to peripheral tissues. The drug crosses the blood–brain barrier and exerts versatile central effects (Uthayathas et al. 2007). Since the NO/cGMP/PDE5 signaling pathway is implicated in the neurobiology of mood disorders, it is possible that sildenafil influences depressive symptoms and the therapeutic potential of antidepressant drugs.

Several previous studies showed that sildenafil produced various effects on the activity of antidepressant drugs in animals. Sildenafil was shown to either reverse (Dhir and Kulkarni 2007a, b; Socała et al. 2012d; Zomkowski et al. 2010, 2012) or enhance (Socała et al. 2012a, b; Socała et al. 2012c) or not to affect (Brink et al. 2008; Socała et al. 2012c) the activity of some antidepressants in the forced swim test in rodents. For this reason, we aimed to extend the studies on other antidepressant drugs, namely sertraline, fluvoxamine, citalopram, maprotiline, trazodone, and agomelatine. The tested compounds vary in their mechanism of action. Sertraline, fluvoxamine, and citalopram belong to the SSRIs; maprotiline is a noradrenaline reuptake inhibitor; trazodone is a mixed serotonin reuptake inhibitor and a 5-HT_2A_/5-HT_2C_ receptor antagonist; while agomelatine is a melatonin receptor agonist and a 5-HT_2C_ receptor antagonist. Although the main mode of action of sertraline, fluvoxamine, and citalopram is the same (i.e., they selectively inhibit the reuptake of serotonin), the drugs within this class show few differences from one another. For example, citalopram is the most selective inhibitor of serotonin reuptake currently available (Bezchlibnyk-Butler et al. 2000), while sertraline is metabolized into desmethylsertraline that shows significantly higher selectivity towards noradrenaline than serotonin transporters. Moreover, sertraline exerts comparatively marked affinity for dopamine transporters whereas fluvoxamine for sigma_1_ receptors (Millan 2006). The results obtained in the present study showed that acute sildenafil treatment potentiated the antidepressant-like action of all of the studied drugs, regardless of their mechanism of action. Despite the fact that trazodone and agomelatine were devoid of anti-immobility action in the forced swim test, the antidepressant-like effect was observed when these drugs were co-administered with sildenafil.

Sildenafil is frequently used as a pharmacological tool for studying the role of the NO/cGMP/PDE5 pathway in the mechanism of action of other drugs. In many studies, sildenafil, injected at a relatively small dose of 5 mg/kg, was shown to abolish the antidepressant-like activity of various compounds, indicating that these substances exert their effects in the forced swim test by decreasing cGMP levels (Almeida et al. 2006; Ghasemi et al. 2008; Ostadhadi et al. 2016; Haj-Mirzaian et al. 2016; Khan et al. 2016). Here, we report that sildenafil injected at higher doses (20–40 mg/kg) did not reverse the anti-immobility effects of antidepressant drugs. On the contrary, it potentiated their action in the forced swim test, which suggests that sildenafil may have opposite effects depending on the dose used. Dual effects of the NO/cGMP pathway modulators have been already reported. The NO donors as well as NO synthase inhibitors can both increase and decrease the duration of immobility in a dose-dependent manner. For instance, l-arginine (a NO precursor) when injected at low doses increased the immobility time, while at higher doses, it decreased the immobility time in the forced swim test (Inan et al. 2004).

Although the forced swim test paradigm is the most widely used animal model for studying antidepressant activity, it has numerous limitations such as false positive or negative results produced by drugs affecting locomotor activity (Petit-Demouliere et al. 2005). In the present study, the anti-immobility effects observed in the forced swim test were not due to the general increase in locomotor activity in mice, as no hyperlocomotion was observed. On the contrary, mice treated with maprotiline and trazodone as well as with their combination with sildenafil exhibited a decrease in locomotor activity. Sedative effects of maprotiline and trazodone were likely related to their ability to block histamine H_1_ receptors (Kanba and Richelson 1986; Millan 2006). A significant decrease in the locomotor activity after joint administration of agomelatine and sildenafil but not agomelatine alone was observed. The changes in the locomotor activity did not interfere with the results obtained from the forced swim test. Nevertheless, it appears that a combination of sildenafil with agomelatine may produce some adverse effects, such as sedation.

The interactions between sildenafil and the studied antidepressant drugs may have pharmacodynamic and/or pharmacokinetic basis. In short, pharmacodynamic interaction occurs when one drug alters the effect of another drug without affecting its concentration in the blood or the site of action, whereas pharmacokinetic drug–drug interaction occurs when one drug changes the concentration of another drug by affecting its absorption, distribution, metabolism, or excretion (Corrie and Hardman 2014). The pharmacokinetic interaction between sildenafil and the studied antidepressants is probable because sildenafil was reported to inhibit the cytochrome P450 isoenzymes, such as CYP1A2, CYP2C9, CYP2C19, CYP2D6, CYP2E1, and CYP3A4 (Hyland et al. 2001), most of which are involved in the metabolism of antidepressant drugs (Spina et al. 2008). In addition, the results of in vitro studies indicate that sildenafil is an inhibitor of drug transporters, such as ABCB1/P-gp, ABCG2/BCRP, ABCC4/MRP4, ABCC5/MRP5, and ABCC10/MRP7, some of which are present in the blood–brain barrier. However, up to date, there are no convincing data confirming that this drug may cause significant interactions with ABCB1/P-gp and ABCG2/BCRP in vivo (Mei et al. 2015). Therefore, to evaluate the potential pharmacokinetic interactions between the studied antidepressant drugs and sildenafil, brain and serum concentrations of antidepressant drugs were determined. Data from pharmacokinetic studies revealed an increased concentration of fluvoxamine, citalopram, and agomelatine in the mouse brain, i.e., at the site of action of antidepressant drugs, after sildenafil co-administration. Additionally, sildenafil elevated serum agomelatine level. The obtained results suggest that the interactions between fluvoxamine, citalopram, or agomelatine and sildenafil could have been pharmacokinetic in nature. The changes in brain concentrations of these antidepressant drugs caused by sildenafil co-administration might have been due to an inhibition of enzymes involved in biotransformation of antidepressants studied or their transport through the blood–brain barrier. No alterations of brain and serum concentrations of sertraline, maprotiline, and trazodone were noted. The increase in antidepressant-like activity of these drugs was most likely related to pharmacodynamic interactions (e.g., at the receptor or signaling level).

Different types of results were expected in the present study, namely the enhancement of the antidepressant activity of the studied drugs by sildenafil co-administration, the reversal of their anti-immobility action, or the lack of any effects. Therefore, we investigated the effect of sildenafil administered at several doses on the action of antidepressant drugs injected at an effective dose. Further studies are required to better characterize the type of interactions between sildenafil and the studied antidepressant drugs as sildenafil injected alone (at a dose of 60 mg/kg) was also shown to produce an anti-immobility action in the forced swim test (Socała et al. 2016). For example, combinations of an ineffective dose of sildenafil with an ineffective dose of antidepressant drugs should be tested to confirm the presence of an additive/synergistic interaction.

Since antidepressant medications often fail to adequately treat severe depression, non-pharmacological interventions for treatment-resistant depression have to be used. ECT remains the most effective form of antidepressant therapy currently available. Despite a broad clinical use of ECT, the mechanism underlying its efficacy is not fully understood (Payne and Prudic 2009). Emerging data suggest that the increase in the expression of brain-derived neurotrophic factor (BDNF) may underlie the antidepressant effect of ECT (Polyakova et al. 2015). Rosen et al. (2003) suggested a significant role of NO in the mechanism of action of ECT. They hypothesized that the involvement of NO in the long-term potentiation, the *N*-methyl-d-aspartic acid (NMDA) receptor activity, the hypothalamic–pituitary axis activity, and the regulation of cerebral blood flow may be critical in ECT’s efficiency (Rosen et al. 2003). As sildenafil elevates BDNF level (Puerta et al. 2010) and, what is more, it works via the NO/cGMP/PDE5 pathway, we decided to investigate its influence on the antidepressant-like efficacy of the repeated ECS in mice. In the first experiment, acute administration of sildenafil produced no changes in the anti-immobility action of the ECS procedure. Therefore, in the next experiment, the influence of subchronic (14 days) treatment with sildenafil was studied. Interestingly, repeated administration of sildenafil dose dependently decreased the antidepressant-like potential of the ECS in mice. Sildenafil is considered to prolong the effects mediated by NO, and the enhancement rather than suppression of the antidepressant-like effect of ECS by sildenafil was expected. The observed effect could have been related to the activation of a negative feedback mechanism within the NO/cGMP/PDE5 signaling pathway. The production of NO may have been downregulated through negative feedback evoked by accumulation of cGMP in response to the prolonged administration of sildenafil (Hotchkiss et al. 2005). Furthermore, discontinuation of the 14-day continuous sildenafil treatment may have led to withdrawal symptoms, such as increased immobility time in the forced swim test. Possible addictive effects of sildenafil use have not been extensively studied as yet. Nevertheless, epidemiologic studies indicated that sildenafil is sometimes abused in a recreational fashion. In addition, animal studies showed rewarding properties of sildenafil in the conditioned place preference paradigm (Tahsili-Fahadan et al. 2006).

The effects observed in the forced swim test were not due to the changes in locomotor activity. In acute studies, no alterations in spontaneous locomotor activity were observed. Unexpectedly, hypolocomotion was noticed in mice subjected to the ECS procedure and injected with either vehicle or sildenafil for 14 days. It is known that repeated i.p. injections can pose a stress risk. Of note, different levels of Fos immunoreactivity after stress induced by repeated i.p. injection and handling in two strains of mice were reported, which suggests that genetic background may also affect behavioral response to the injection stress and habituation procedure (Ryabinin et al. 1999). Based on our observation, it seems that the ECS procedure and repeated i.p. injections may produce different behavioral changes in comparison with ECS treatment and acute i.p. injections. Further studies are required to fully understand the mechanism underlying the observed phenomenon. Nevertheless, since the decreased locomotor activity was observed in animals subjected to the ECS procedure and injected with sildenafil as well as in animals mice subjected to the ECS procedure alone, the changes in locomotion did not interfere with the results from the forced swim test.

To conclude, the present findings demonstrate for the first time that sildenafil augments the activity of several antidepressant drugs in the forced swim test in mice. No changes in brain concentrations of sertraline, maprotiline, and trazodone suggest that the interactions between sildenafil and these drugs were pharmacodynamic in nature. In contrast, an increased activity of fluvoxamine, citalopram, and agomelatine was related, at least in part, to their pharmacokinetic interactions with sildenafil. However, caution should be taken in the extrapolation of our results to humans because the biotransformation of the studied antidepressants may vary between mice and humans. Moreover, we showed that acute treatment with sildenafil did not influence the antidepressant-like effect of the ECS procedure in mice, while subchronic (14 days) treatment with this drug abolished the anti-immobility action of this procedure in mice. Further studies are required to develop a safe and effective antidepressant treatment in patients taking sildenafil.

## Abbreviations

BDNF: Brain-derived neurotrophic factor
cGMP: Cyclic guanosine 3′,5′-monophosphate
ECS: Electroshock stimulation
ECT: Electroconvulsive therapy
HPLC: High-performance liquid chromatography
i.p.: Intraperitoneally
IS: Internal standard
NO: Nitric oxide
PDE5: Phosphodiesterase type 5
SEM: Standard error of the mean
SSRIs: Selective serotonin reuptake inhibitors
